# Recreating the human brain: Are assembloids merely descriptive models?

**DOI:** 10.3389/fncel.2026.1787173

**Published:** 2026-03-27

**Authors:** Yara Izhiman, Charitha Anamala, Eric A. Nauman, Volha Liaudanskaya

**Affiliations:** 1 Department of Biomedical Engineering, College of Engineering and Applied Science, University of Cincinnati, Cincinnati, OH, United States; 2 Department of Mechanical and Materials Engineering, College of Engineering and Applied Science, University of Cincinnati, Cincinnati, OH, United States; 3 Neuroscience Graduate Program, University of Cincinnati, College of Medicine, Cincinnati, OH, United States

**Keywords:** assembloids, functional models, glial regulation, neurodevelopment, neurological disease

## Abstract

Assembloids, engineered fusions of region-specific brain 3D constructs, have emerged as powerful platforms to study neurodevelopment and neurological diseases. Unlike first-generation organoids, assembloids enable direct modeling of interregional communication, allowing investigation of higher-order brain functions that depend on circuit-level interactions. Over the past 5 years, rapid advances in human-derived assembloid systems have demonstrated their ability to recapitulate key features of human brain organization, including long-range projection formation, region-specific signaling, neurovascular coupling, and progressive network dysfunction. The primary application of assembloid modeling remains the study of neurodevelopment, specifically focusing on mapping biological mechanisms driving the human brain development. Another major application of assembloids is the study and modeling of neurological diseases. Recent studies have integrated multiple neural regions, alongside vascular and glial components, and disease-relevant genetic backgrounds to recreate circuit-level interactions underlying pathology. These approaches have further highlighted the importance of neuroglial interactions in shaping development, connectivity, and disease progression in the human brain. Across models and disease contexts, a consistent theme has emerged: pathological phenotypes arise primarily from disrupted intercellular communication rather than isolated cellular and more purely neuronal, defects. Despite these strengths, current assembloid platforms remain limited by incomplete maturation, variability in reproducibility, and challenges in modeling long-term disease trajectories. Together, existing evidence positions assembloids as a promising next-generation platform for studying human brain development and neurodegeneration, while highlighting the need for continued refinement to improve physiological relevance as model complexity increases.

## Introduction

Modeling the human central nervous system (CNS) is an intricate process that relies on balanced cellular interactions in tandem with the cytoarchitecture and system functionality. Brain organoids, three-dimensional self-organizing structures derived from stem cell cultures, have been a leading platform in replicating histological features of the human brain and modeling neural disorders (Wu and Nowakowski, 2025; Chen et al., 2020; Makrygianni and Chrousos, 2021). Brain organoids have been used to replicate the highly ordered processes driving cellular differentiation, organization, and communication, furthering our understanding of the mechanisms driving human CNS development. These platforms, however, lack a reproducible topographic cellular organization, which limits their modeling application. In addition, grasping the intricate neural networks and interregional brain interactions using these mono-source organoids remains challenging. This limitation drove the innovation of assembloids, which are defined here as fusions of 3D constructs that integrate multiregional and multicellular interfaces, to explore regional brain patterning and complex interregional interactions (Birey et al., 2017; Xiang et al., 2017; Bagley et al., 2017). In recent years, assembloids have grown into a viable tool to characterize CNS inter-regional function.

The ability to create region-specific, vascularized, and neuroglial assembloids allows for higher fidelity modeling of human brain development and neurodegenerative processes. Given the distinct cellular environments and progenitor domains, successful brain-aggregate modeling requires precise control of region-specific cellular differentiation. Recent efforts in the field have resulted in various fusion combinations of assembloids, such as dorsal-ventral forebrain (Birey et al., 2017, 2022; Samarasinghe et al., 2021; Pipicelli et al., 2023; Walsh et al., 2025), cortical-hippocampal (McCrimmon et al., 2025), cortical-thalamic (Shin et al., 2024; Kim et al., 2024), cortical-diencephalic (Kim J. I. et al., 2025), cortical-striatal (Miura et al., 2020), sensory-spinal-diencephalic-cortical (Kim J. I. et al., 2025), vascularized-cerebral (Dao et al., 2024), and cortical-motor assembloids (Andersen et al., 2020). These different compositions enable the modeling of a wide spectrum of neurodevelopmental and neurodegenerative diseases, such as autism spectrum disorder (ASD), epilepsy, schizophrenia, Huntington’s disease (HD), Parkinson’s disease (PD), and Alzheimer’s disease (AD). The promise of assembloids in neurodevelopment characterization and neurological disease modeling inspired this review, which synthesizes current advances, established knowledge, and emerging directions in the field.

## Assembloids for neurodevelopment characterization

**FIGURE 1:**
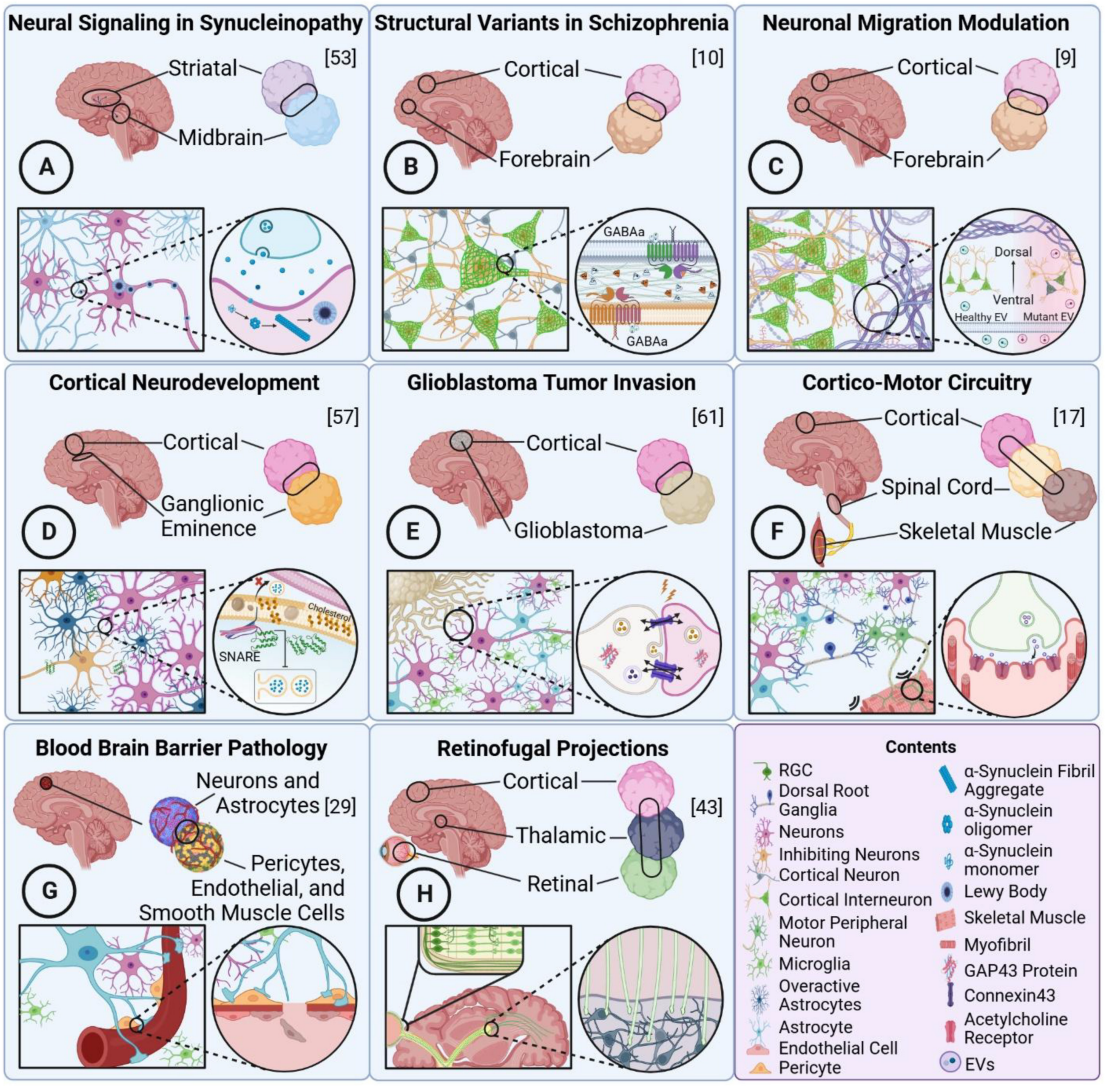
An overview of key reviewed assembloid platforms. An illustration of the modeled brain region, interregional fusion of 3D structs, and specific biological mechanism modeled through the assembloid for eight notable platforms. **(A)** Striatal-midbrain model of synucleinopathy and dopaminergic vulnerability in PD, **(B)** cortical-forebrain model of Schizophrenia-associated interneuron structural variants, **(C)** cortical-forebrain model of impaired ventral to dorsal interneuron migration in PH, **(D)** cortical-ganglionic eminence model of APOE4 effects on neurodevelopment and network stability, **(E)** glioblastoma-cerebral model of tumor invasion and microtubule formation, **(F)** cortical-spinal cord-skeletal muscle model of cortico-motor circuitry integration and skeletal muscle contraction, **(G)** cortical blood brain barrier model of cerebral cavernous malformations and mural cell breakdown, and **(H)** cortical-thalamic-retinal model of the visual pathway in terms of retinal ganglion cellular outgrowth and projection.

Assembloids, engineered fusions of region, cell, and lineage-specific tissues (Figure 1), are reshaping how neurodevelopment is studied by moving beyond cell-autonomous toxicity toward circuit and niche-dependent disease mechanisms (Wu and Nowakowski, 2025; Kanton and Pasça, 2022; Onesto et al., 2024). The greatest advantage of the assembloid platform lies in mimicking cell-to-cell interactions along with inter-region neural function and network mapping. This is especially critical for unveiling the systemized biological mechanisms during early-stage neural development. Historically, mouse models governed the study of these processes, providing critical insights into mammalian brain neurodevelopment (Dehorter and Del Pino, 2020; Boroviak et al., 2018; Leung and Jia, 2016; Damianidou et al., 2022). While mouse models provided the foundation of our current knowledge about the human brain, the intricacy of human-specific processes was only captured through *in vitro* human modeling (Nakajima and Schmitt, 2020). Hence, assembloids serve as mechanistic platforms that enable the recreation and controlled investigation of key developmental processes of the human brain, such as cross-regional neuronal migration, neuroglial cellular interactions, and functional connectivity.

In this context, assembloid systems that fuse discrete 3D constructs interfaces provide a powerful framework to study region-specific regulatory variability, neuronal migration, axonal projection, and the emergence of interregional connectivity. Specifically, analysis of axonal outgrowth regulatory processes enables characterization of functional neuronal network arrangement, termed circuit-level organization (Onesto et al., 2025). Circuit-level analysis examines how neuronal groups establish directional connectivity, coordinate activation patterns, and integrate spatially. Consistent with this, the human floor plate assembloid acts as a neuronal network organizer during early development by regulating guidance cues, cellular interactions, and axonal projection (Onesto et al., 2025). In parallel, studies have demonstrated region-specific biases in axon targeting (Birey et al., 2022; McCrimmon et al., 2025; Cassel de Camps et al., 2024), wherein axonal projections originating from midbrain organoids preferentially innervate other midbrain organoids within organized assembloids (Cassel de Camps et al., 2024). These findings highlight the existence of region-dependent rules governing neuronal connectivity and underscore the selective nature of organoid fusion and integration. By serving as experimentally tractable systems, assembloid platforms are able to probe genetic and molecular perturbations contributing to pivotal neurodevelopmental mechanisms that cannot be accessed in isolation.

The vascularization and maturation of the blood-brain barrier (BBB) is another critical stage of human brain development, as this protective barrier regulates CNS function and protects the brain against developmental abnormalities and disorders (Saili et al., 2017). The human BBB exhibits molecular and functional properties only partially replicated in animal or conventional *in vitro* models, challenging our ability to study its physiology and the mechanisms driving its dysfunction. Conventional models present limited in capturing the bidirectional cellular interaction, multicellular architecture, 3D geometry, as well as dynamic function in the BBB. To address these limitations, Dao et al. (2024) introduced a BBB assembloid model (Figure 1G) that incorporates vascular, perivascular, and neuronal components to mimic the molecular, cellular, anatomical, and functional characteristics of the BBB. This BBB model permits unique neuro-vascular interaction mapping, which is theorized to govern various neurodegenerative diseases by controlling where and how neurons get vascular support, protection, and clearance (Dao et al., 2024). Given that the assembloid model originates from patient-derived induced pluripotent stem cells, validating patient-specific phenotypic features is critical to differentiate authentic disease-related mechanisms from generic developmental variation. Upon extensive validation, patient-specific BBB assembloids have the potential to replicate neuro-vascular dynamics and to serve as a screening tool for various neurovascular pathologies.

In terms of cortical dynamics, thalamocortical pathway development is considered a focal system in processing sensory information and modulating cognitive function (Sydnor et al., 2025). While the thalamocortical pathway exhibits stronger connections with age, the regulatory processes underlying its maturation and the pathway-specific differences across development timing are not well defined. This gap drove the design of a thalamocortical assembloid model that integrates diencephalic organoids with cortical organoids (Kim et al., 2024). To validate functional thalamocortical pathway development in these assembloids, Kim et al. (2024) induced changes in thalamic activity through a CACNA1G gene-variant. Beyond neurodevelopment, this model could serve as a tool for modeling neuropsychiatric disorders such as schizophrenia, where the disease is associated with a functional dysconnectivity in the thalamocortical pathway (Roy et al., 2021).

The corticostriatal pathway is a subsequent forebrain circuit of interest that regulates motivated behaviors, movement control, and decision-making (Shepherd, 2013). This pathway is associated with neurodevelopmental disorders, such as ASD, obsessive-compulsive disorder, and schizophrenia (Walsh et al., 2025; Miura et al., 2020; Shepherd, 2013). These disorders may stem from a corticostriatal abnormality during early developmental periods, despite their symptoms becoming apparent at later stages (Li and Pozzo-Miller, 2020; Cainelli and Bisiacchi, 2022). To characterize the abnormalities in the corticostriatal circuitry, Miura et al. (2020) developed a corticostriatal assembloid and traced cortical axonal projections into striatal organoids. This patient-derived neurodevelopmental disorder assembloid model additionally captured the neuronal defects associated with the deletion of chromosome 22q13.3 on calcium activity. In parallel, Meng et al. (2023) developed a CRISPR screening assembloid model to evaluate neurodevelopmental genetic involvement in abnormal cortical interneuron development. Beyond describing neurodevelopmental trajectories, these platforms can capture emerging markers of neurodevelopmental dysfunction, positioning them as early detection tools.

Building upon large-scale pathway organizations, such as the thalamocortical and corticostriatal pathways, it is important to consider the local neuroglial interactions that govern pathway connectivity and functionality. In the context of neocortical development, outer radial glia are key drivers of cortical expansion through the development of an enlarged outer subventricular zone (oSVZ) (Florio and Huttner, 2014). Walsh et al. (2024) highlighted the importance of promoting outer radial glia emergence through leukemia inhibitory factor treatment. Specifically, their work focused on producing cortical assembloids with an expanded oSVZ, achieving a more physiologically accurate cortical architecture (Walsh et al., 2024). Beyond cellular architecture, this model expands cellular and progenitor complexity to accurately recapitulate the human cortical microenvironment, enabling precise analysis of radial glia organization, oSVZ formation, and early circuit maturation. These regulatory architectures highlight that only human−derived models can capture neurodevelopment with fidelity, making downstream functional and disease analysis more human-relevant.

Functionality of brain models is another domain central to understanding neurodevelopment patterns and abnormalities. More specifically, models that capture the ascending and descending pathways in the human brain can provide insight into generalized and patient-specific developmental patterns. The corticomotor circuitry is a species-specific function describing movement coordination and muscular control, which can unlock an in-depth understanding of motor disorders and functional motor control. Andersen et al. (2020) derived a hindbrain-spinal cord assembloid with functional motor circuitry (Figure 1F). In cojoining a cortico-motor assembloid with a skeletal organoid, they created a three-component system that drove muscle contraction, verifying a functional neural circuit development. To validate neural signal transmission, Son et al. (2022) proposed an electrophysiological monitoring approach of their cerebral-motor assembloid encompassing the cerebellum, hypothalamus, spinal cord, and motor neuron spheroids. Assessing motor neurons signal transmission makes this model functionally, not just anatomically, relevant and reveals early circuit–level defects in neurodevelopmental and motor disorders. The application of these corticomotor models goes beyond exploring neurological diseases and developmental mechanisms, with a potential to examine unique corticomuscular control dynamics and neuroplastic adaptations following a motor disease or injury.

Somatosensory circuitry is an inseparable part of the corticomotor system, focused on conveying peripheral sensory feedback for adaptive motor control. Through a four-part assembloid model encompassing cortical, thalamic, spinal, and somatosensory organoids, Kim J. I. et al. (2025) developed a functional model of the major components of the ascending somatosensory pathway. The distinctive characteristic of the model was highlighted by the role of SCN9A in regulating neural synchronous activity. SCN9A is a gene regulating the activation of voltage−gated sodium channel Nav1.7, which is essential for nociception (Deng et al., 2023). Nociception functions early in development as a human-specific driver of synchronized activity and circuit assembly across sensory, spinal, thalamic, and cortical domains, making it a key organizer of neurodevelopment. With pain and sensory disorders stemming from disrupted signal propagation, this spinothalamic-tract assembloid structure can assess the long-range directional projections underlying sensory disorders, to expose defects in neuronal synchrony and sensory-relay that simple models cannot capture (Kim J. I. et al., 2025).

Retinofugal projections, long-range axons of retinal ganglion cells (RGCs) extending to thalamic and cortical targets, are another pivotal circuitry for establishing the functional visual system during development (Chen et al., 2025; Hayes et al., 2026; Fligor et al., 2021). Successful development of these pathways enables proper visual processing, circadian regulation, and non–visual behaviors, while their disruption contributes to optic neuropathies such as glaucoma and multiple–sclerosis–related optic neuritis (Hayes et al., 2026). To characterize the developmental roles of retinofugal projections, retinal-brain assembloids have been proposed to study axon guidance, neurotrophic support, and glial interactions, revealing how intrinsic RGC programs and extrinsic brain cues work jointly during early neurodevelopment (Chen et al., 2025; Hayes et al., 2026; Fligor et al., 2021). To overcome limitations of standalone retinal organoids lacking brain targets, Fligor et al. (2021) developed a retinal-thalamic-cortical assembloid (Figure 1H) that mimics the developing visual pathway. Interestingly, this tri-partite assembloid revealed that the thalamic identity provides a more competent retinorecipient environment, emphasizing its role in guiding RGC guidance and growth. In parallel, Chen et al. (2025) introduced a vascularized-retinal assembloid to reconstruct the inner blood-retinal barrier and allow for immune-responsive testing of inflammatory stimuli. While the model captures the neurovascular and neuroimmune interactions during retinal development, its application extends to the assessment of vascular-targeted therapeutics, blood-retinal barrier permeability, and neuro-immune crosstalk, thereby extending utility to pharmacodynamics, toxicology, and precision-medicine studies. In an effort to mitigate significant phenotype variability and facilitating clinically viable platforms, Hayes et al. (2026) fused retinal organoids and oligodendrocyte-rich cortical organoids in the absence of animal-derived reagents. Beyond elucidating developmental biology, this model recapitulates RGC axon extension and myelination processes further allowing it to serve as a translational pipeline for drug screening and cell-therapy manufacturing by providing a fully human, myelinated visual-pathway model.

Although human-derived assembloids hold increasing promise for neurodevelopmental characterization, their application remains at an early stage. Current assembloid platforms capture only limited aspects of neurodevelopment and are largely restricted to early developmental windows, reflecting ongoing challenges in achieving prolonged maturation and extending these systems toward later developmental stages. As a result, modeling adult neurogenesis and late-stage neurodevelopmental processes remains difficult. Nevertheless, despite these limitations, assembloids offer substantial value as disease modeling and detection platforms, particularly for investigating early-emerging pathological mechanisms in a controlled, human-relevant context. Table 1 summarizes the reviewed neurodevelopmental assembloid platforms, outlining their applications, key contributions, limitations, and future directions.

**TABLE 1:** Summary of the reviewed assembloid platforms.

Study	Model type	Assembloid components	Main objective	Contributions/findings	Limitations/future directions
Kim et al., 2024	Thalamocortical assembloid	Diencephalic organoids fused with cortical organoids	Study thalamocortical pathway development and psychiatric disease risk	CACNA1G variants alter calcium currents, circuit synchrony, and long-range connectivity relevant to schizophrenia and seizure disorders.	Thalamic nuclei are simplified, and sensory input is absent. Future models should incorporate biomechanical cues and patterned extracellular matrices.
Miura et al., 2020	Corticostriatal assembloid	Cortical organoids fused with striatal organoids	Model forebrain circuit development and activity-dependent maturation	Demonstrating functional cortico-striatal projections, MSN maturation, and disease-associated calcium signaling defects.	Cellular diversity is limited due to the absence of vasculature and immune cells. Future studies should integrate microglia and stiffness-matched scaffolds.
Walsh et al., 2024	Expanded cortical assembloid (oRG/oSVZ model)	Cortical organoids treated with LIF and optionally combined with LIF-producing pericytes	Model human-specific neocortical development	LIF-STAT3 signaling induces outer radial glia expansion and formation of an oSVZ-like progenitor zone resembling the fetal human cortex.	Long-range circuitry is not modeled. Future work should investigate the role of extracellular matrix tension and mechanical forces in cortical expansion.
Andersen et al., 2020	Cortico-motor assembloid	Cortical organoids assembled with spinal cord organoids and skeletal muscle spheroids	Reconstitute functional motor circuitry	Cortical activation propagates through spinal neurons and induces robust skeletal muscle contraction.	Sensory feedback and vascularization are absent. Future models should integrate afferent inputs and perfusable tissue scaffolds.
Chen et al., 2025	Vascularized-retinal assembloid	Vascular organoids fused with retinal organoids in a PDMS V-bottom microwell	Long-term human retinal model exhibiting inner blood-retinal barrier properties	Demonstrating intraretinal vessels with pericyte coverage alongside mature photoreceptors neurovascular unit architecture resembling inner blood-retinal barrier.	Model does not incorporate hemodynamics, limiting its clinical translatability. Future work should examine the mechanistic pathways linking retinal development and vascularization.
Hayes et al., 2026	Retinofugal assembloid	Retinal organoids fused with oligocortical organoids	A xenobiotic-free human-relevant retinofugal assembloid that models development	Establishing a xenobiotic-free model. Demonstrating retinofugal polarity through unidirectional extension of RGC axons. Supporting myelinating glia interactions.	The model exhibits limited functional characterization. Future work should explore the clinical translatability of this platform for drug testing and disease screening.
Fligor et al., 2021	Retinal-thalamic-cortical assembloid	Tri-assembloid combined retinal organoids with cortical and thalamic organoids	Model RGC axon outgrowth and brain integration and reconstruct the retinothalamocortical pathway	Thalamic organoids promote significant RGC axon recruitment. Tri-assembloid recapitulated multiple nodes of the visual pathway.	Model is limited in capturing functional retinotopic mapping. Future work should define regional patterning and expand this model into a disease assessment platform.
Son et al., 2022	Electrophysiological brain-spinal cord assembloid (eBSA)	Cerebral organoids coupled with motor neuron spheroids on multielectrode arrays	Monitor neurochemical-driven signal transmission	Caffeine induces excitatory signal propagation from cerebral organoids to motor neurons, which is detected as increased spiking activity.	Muscle output is not included, and spinal circuitry is simplified. Future systems should incorporate synaptic relays and contractile readouts.
Wu et al., 2024	Striato-nigral assembloid (HD)	Striatal organoids fused with nigral organoids	Model basal ganglia circuit degeneration	Mutant huntingtin disrupts medium spiny neuron projections and long-range circuit integrity.	Vascular and immune components are absent, and late-stage degeneration is limited. Future work should incorporate inflammatory and aging-related factors.
Tran et al., 2025	Striatal-midbrain assembloid (PD)	Striatal organoids fused with dopaminergic midbrain organoids	Model α-synuclein propagation	The model demonstrates retrograde α-synuclein spread, Lewy-like inclusions, and dopaminergic vulnerability.	Disease progression is incomplete. Future models should include chronic degeneration and neuroimmune interactions.
Meyer-Acosta et al., 2025	Cortical-GE assembloid (AD-relevant)	Cortical organoids fused with ganglionic eminence organoids	Study APOE4 effects on early brain development	APOE4 induces premature differentiation, excitatory-inhibitory imbalance, and early network instability.	Amyloid and tau pathology are not modeled. Future work should integrate aging cues and late-stage Alzheimer’s disease features.
Dao et al., 2024	Brain-vessel assembloid (BBB/CCM)	Brain organoids fused with blood vessel organoids	Model BBB disease and cerebral cavernous malformations	The assembloid recapitulates cavernoma-like vascular malformations, barrier breakdown, and mural cell loss.	Systemic circulation is absent, and lesion expansion is limited. Future work should employ perfusion-based systems.
Kim J. et al., 2025	Glioblastoma-cerebral organoid assembloid	Patient-derived GBM tumoroids fused with cerebral organoids	Model tumor invasion and the brain tumor microenvironment	The model captures real-time single-cell and collective invasion, tumor microtube formation, and neuron-guided adaptation.	Immune interactions and therapy-driven evolution are absent. Future studies should incorporate immune cells and treatment pressure.
Walsh et al., 2025	Forebrain assembloid (PVALB + interneuron model)	Dorsal forebrain (cortical) organoids fused with ventral forebrain (MGE-patterned) organoids	Generate bona fide fast-spiking PVALB + cortical interneurons and model schizophrenia-associated structural variants	Established a model that produces molecularly and electrophysiologically validated PVALB+/LHX6 + fast-spiking interneurons within ∼120 days. Schizophrenia-associated structural variants disrupted interneuron migration, maturation, and γ-band oscillatory network activity.	Long-term maturation remains limited relative to adult cortex. Future work should examine later-stage network integration and broader circuit-level dysfunction across additional psychiatric genotypes.
Pipicelli et al., 2023	Dorsoventral cerebral assembloid (LGALS3BP mutation model)	Dorsal cortical organoids fused with ventral forebrain organoids (isogenic LGALS3BP mutant and control lines)	Model periventricular heterotopia–associated cortical malformation and interneuron migration defects	LGALS3BP mutation induced ventral-to-dorsal identity shifts and impaired interneuron migration. Altered extracellular vesicle cargo contributed to non–cell-autonomous defects, with partial rescue observed upon exposure to wild-type vesicles.	Does not model full cortical layering or long-range circuitry. Future models should examine later developmental stages and broader microenvironmental contributions to cortical malformation progression.
Maity et al., 2025	Glioblastoma brain-on-a-chip microfluidic model	Patient-derived GBM tumoroids integrated into a perfusable microfluidic platform incorporating vascular-like channels and brain tissue context	Model glioblastoma invasion and therapeutic response under controlled perfusion conditions	Enables real-time monitoring of tumor invasion under physiologically relevant oxygen, nutrients, and shear stress gradients. Captures BBB-associated drug transport constraints and microenvironment-driven treatment resistance not observed in static systems.	Immune components and long-term tumor evolution remain limited. Future integration of immune cells and patient-specific profiling could enhance translational and diagnostic applications.

## Assembloids for neurological disease modeling

Neurological disease is often conceptualized in terms of cell-type-specific pathology, such as neuronal degeneration, tumor cell proliferation, or aberrant neurodevelopment. Yet many disorders ultimately reflect disruptions in neural circuitry, tissue architecture, and multicellular communication. Across neurodegenerative disorders, malignant brain tumors, and congenital malformations, pathology is increasingly recognized as a network and microenvironment-level process. These disease-related changes reshape axonal projections, synaptic organization, vascular interactions, and glial support systems, urging for platforms that can recapitulate multicellular connectivity and functionality (Wu and Nowakowski, 2025; Chen et al., 2020; Kanton and Pasça, 2022).

Neurodegenerative pathologies are often associated with an abnormal aggregation and accumulation of proteins in the CNS, termed proteinopathies (Walsh et al., 2025). One such pathology is Huntington’s Disease (HD), which is characterized by Polyglutamine (polyQ) aggregation, an expanded huntingtin protein resulting from CAG repeat expansion in the HTT gene (Paulson et al., 2017). This polyQ proteinopathy disrupts cortico-striatal and striatal output pathways within the basal ganglia, leading to progressive degeneration and dysfunction of medium spiny neuron-centered circuits (Wu et al., 2024). The striato-nigral assembloid model established by Wu et al. (2024) captured these circuit vulnerabilities by assessing deficits in medium spiny neuron projections along the basal ganglia axis. In addition to modeling the cellular mechanism in HD, the striato-nigral assembloid can function as a therapy assessment tool to examine circuit interventions in terms of axon projection stability and outgrowth restoration. Hence, this functional assembloid model demonstrates that preservation of circuit wiring, projection stability, and intercellular support provides an early and mechanistically relevant basis for assessing circuit-targeted therapies.

Synucleinopathies are another class of proteinopathies characterized by alpha-synuclein (α-syn) aggregation in neuronal and glial cells that drives motor deficits, such as in Parkinson’s Disease (PD) (Li and Li, 2024; Xu et al., 2025). In PD, accumulation of α-syn in the form of Lewy bodies is considered a hallmark of disease pathogenesis, given its modulation of CNS cellular mechanisms (Xu et al., 2025). To shift experimental investigation away from terminal degeneration and toward earlier disease phases, Tran et al. (2025) introduced a striatal-midbrain assembloid model (Figure 1A) to recapture basal ganglia circuits with α-syn propagation in nigrostriatal and striatonigral pathways. This assembloid model captures the accumulation of misfolded α-synuclein and its effect on dopaminergic neuronal projection and synaptic transmission. It also examines the neuroprotective pathways and the role glial cells play in regulating neuronal uptake of α-synuclein. Accordingly, a central advantage of employing assembloids in disease modeling is evaluating spread and functional disruption in controlled circuity (Kanton and Pasça, 2022; Onesto et al., 2024; Ouaidat et al., 2025).

Similar to synuclein-driven circuit disruption in PD, protein aggregation also underlies AD, where early imbalance in amyloid-β production and clearance is increasingly considered a driver of network dysfunction rather than merely a late-stage consequence of neurodegeneration (Hardy and Selkoe, 2002). A critical regulatory gene for amyloid-β clearance is APOE4 (Poblano et al., 2024). APOE4 reduces excitatory cortical neurons, increases inhibitory GABAergic neuron differentiation, promotes aberrant gliogenesis, and induces premature or dysregulated network activity, which leads to impaired circuit organization (Meyer-Acosta et al., 2025). Considering systems-level disease evaluation of AD, Meyer-Acosta et al. (2025) developed a cortical and ganglionic eminence assembloid model (Figure 1D), where ganglionic eminence is an embryonic structure guiding neuronal proliferation, to evaluate how APOE4 reshapes early neurodevelopmental trajectories (Dias et al., 2025). Because this system reconstructs long-range interneuron migration and functional synaptic coupling, it enables longitudinal measurement of early excitation/inhibition imbalance and network hyperexcitability as quantifiable biomarkers of APOE4-driven risk before amyloid deposition. Overall, assessing protein and genetic encryption in assembloids unveiled the detailed neuroglial interactions underlying neuronal survival, death, and disease presentation (Meyer-Acosta et al., 2025; Li et al., 2025). The platform could therefore support real-time electrophysiological and cellular screening to determine whether candidate therapeutics normalize circuit maturation dynamics, offering a developmentally informed strategy for early intervention rather than late-stage plaque targeting.

Extending the utility of assembloids to neuropsychiatric diseases, Walsh et al. (2025) developed a human forebrain assembloid model (Figure 1B) that functionally fuses dorsal and ventral forebrain organoids to capture early dorsal-ventral forebrain circuit assembly to study schizophrenia. CRISPR-engineered schizophrenia-associated structural variant assembloids were compared to healthy controls to assess their ability to model early circuit assembly, interneuron migration, transcriptional identity, and real-time γ-band network activity (Walsh et al., 2025). This model’s capacity to reveal early electrophysiological and transcriptional abnormalities could support applications in early-risk stratification and the development of preventative interventions to restore network stability before symptom onset. For example, it could assess disease-related effects at multiple stages of development to pinpoint how defects at earlier stages add up to abnormal inhibitory control and disrupted γ-band-like activity. The application of this platform could extend to other neuropsychiatric diseases such as epilepsy and autism spectrum disorders, which are linked to 22q11.2 and 15q13.3 deletions. Most excitingly, the CRISPR-based isogenic engineering strategy is readily adaptable to modeling additional neurodevelopmental and psychiatric disorders characterized by circuit-level dysfunction, broadening the utility of this platform for precision neuropsychiatric research.

Aside from protein aggregation disorders and neuropsychiatric disorders, assembloid systems have also been applied to model neurodevelopmental brain disorders, like Periventricular Heterotropia (PH), for example. PH is a neurodevelopmental cortical malformation marked by ectopic neuronal clusters lining the lateral ventricles due to failed neuronal delamination and migration during corticogenesis, with pathogenic variants in LGALS3BP identified in affected individuals (Kyrousi et al., 2021). Pipicelli et al’s (2023) LGALS3BP mutant dorsal-ventral forebrain assembloid (Figure 1C) exhibited disrupted dorsoventral patterning, reduced interneuron specification, and impaired migration dynamics compared to healthy assembloids. Most importantly, they found evidence that altered non-cell-autonomous extracellular vesicle (EV) signaling contributed to these defects and that some aspects of the phenotype were even partially reversible (Pipicelli et al., 2023). The partial rescue via wild-type EVs is particularly significant, as it demonstrates that PH-associated defects arise from modifiable microenvironmental signaling disturbances, highlighting extracellular communication as both a mechanistic driver and a potential therapeutic target. By enabling mechanistic testing of extracellular signaling and rescue within a multicellular context, this system demonstrates how assembloids can move beyond descriptive modeling to support therapeutic targeting, biomarker discovery, and precision diagnostics in neurodevelopmental disorders.

While assembloid platforms have been widely applied to study neurodevelopmental and neurodegenerative disorders, their relevance can also extend to malignant brain disease. Glioblastoma pathology is driven by aberrant stemness programs and invasive mesenchymal-like transitions within the tumor microenvironment (Meyer-Acosta et al., 2025; Dias et al., 2025; Li et al., 2025). Building on these mechanistic studies, Kim J. et al.’s (2025) patient-derived glioblastoma assembloid (Figure 1E) reconstructs tumor-brain and perivascular interactions in a physiologically relevant 3D context that remains unattainable in conventional organoid systems. This platform faithfully reproduces hallmark invasive behaviors, covering diffuse tumor infiltration, directional migration, and tumor microtube formation (Kim J. et al., 2025). In the context of an “immune cold” cancer like glioblastoma, these types of models are invaluable in developing targeted treatment options (Liu et al., 2024). Moving beyond the conventional assembloid model, brain-on-a-chip systems introduce an added layer of physiological relevance through controlled microfluidic perfusion. Unlike static organoids or spheroid models, Maity et al.’s (2025) glioblastoma on a chip allows for the generation of oxygen and nutrient gradients, and real-time monitoring of tumor invasion and therapeutic response (Lee et al., 2025). Critically, they address a major limitation in glioblastoma modeling, such as the inability of conventional systems to capture BBB-mediated drug transport and vascular shear stress, both of which strongly influence treatment efficacy. Building on this framework, future glioblastoma assembloid models should expand cellular complexity to more fully represent the tumor microenvironment. As such, beyond serving as experimental models of tumor biology, glioblastoma assembloids have the potential to act as translational tools that bridge mechanistic insight with next-generation diagnostic and therapeutic development.

Despite the advancements and promise of neurodegeneration modeling using human-derived assembloids, the field remains constrained by incomplete maturation, variability in model efficiency, and imperfect BBB functionality. The current assembloid modeling ability encompasses regional models such as forebrain, hindbrain, cortical-motor, and cortical-sensory assembloids, which remain incomplete representations of the human brain. These challenges directly limit efficient neurodegeneration and neurological disease modeling, where late-stage phenotypes and chronic stress are central. Table 1 summarizes the key contributions, limitations, and future applications of the reviewed neurological disease assembloid platforms.

## Considerations in future directions

Consciousness can be argued as the sensory interaction with the world, and in biological systems, it is supported by a functional neural cytoarchitecture capable of somatosensory processing, neural firing, and electrical synchrony (Croxford and Bayne, 2024; Kataoka et al., 2025; Lavazza, 2020). While an argument against consciousness could be made when evaluating disembodied neural organoids, neural assembloids challenge the philosophical embodiment argument for consciousness. The embodiment theory argues that a history of embodiment and sensorimotor interaction with the external environment is necessary to support consciousness, which is the case for sensorimotor assembloid platforms (Croxford and Bayne, 2024). These platforms, while not developmentally mature, are expanding the potential to develop sensory and motor functionality, moving beyond simple descriptive models. The expansion to fully functional assembloids would encompass sensory, emotional, and cognitive processing, all of which are indicative of a functional-living entity. Such functionality requires transparency about maturation state and functional complexity to better describe potential ethical ambiguity (Wu and Nowakowski, 2025; Kanton and Pasça, 2022; Onesto et al., 2024). To this day, the lack of full functionality of human brain assembloids in the field mitigates a primary ethical concern, as current assembloids remain developmentally immature.

Human brain assembloid platforms, while revolutionary in modeling patient-specific physiology, remain limited in capturing the architectural similarity to the human brain, especially in terms of network mapping, neuro-immune interactions, and mature vasculature (Table 1). Specifically, the reproducibility of network mapping remains challenging, further urging pattern identification investigations to evaluate drivers of different fusion approaches. Standardization of assembloid development unlocks potential clinical applications for functional patient-derived brain assembloids, serving as a drug testing platform for neural diseases such as Huntington’s (Wu et al., 2024), a brain-delivery drug screening tool (Castagnola et al., 2023), a brain tumors treatment-response evaluating tool (Sinha et al., 2024; Maity et al., 2025), and a genetic screening tool for neural disease risk and neuronal defects (Walsh et al., 2025). Though still emerging, assembloids also offer high-fidelity assessments of drug efficacy, resistance, and delivery by recapitulating personalized microenvironment features such as the BBB and relevant spatiotemporal gradients.

A critical gap in current assembloid work remains the adaptation of different mechanical factors within the assembloid microenvironment (Table 1). Mechanical factors, such as the extracellular matrix’s contractility, stiffness, axial forces, structural organization, viscoelasticity, and fluid shear stress, drive different cellular differentiation responses (Liaudanskaya et al., 2023; Zhang et al., 2025, 2026; Gentleman et al., 2004, 2007; Lewus and Nauman, 2005). Such mechanistically driven responses emphasize the need to expand research into the role of extracellular matrix mechanics in assembloid mechanical properties and structural models. For instance, microenvironment contractility decreases cellular proliferation and growth in 3D constructs (Ahn et al., 2024), which is an important consideration when designing matrices that guide morphogenesis. Hence, driving specific microenvironment mechanical investigations that recapitulate the architecture of the human brain may position assembloids as preventative tools to screen premature hallmarks of neural diseases.

A persistent limitation in fostering anatomically relevant assembloids is capturing the interregional cellular architecture. Current assembloid platforms are most focused on the fusion of mono-source constructs, which present a limitation in accurately describing neuronal-glial interactions within the 3D constructs driving the assembloid structure. Mono-source 3D constructs develop within an impoverished cellular environment that lacks intercellular signaling, co-maturation, and circuit-level refinement that shape the complexity of the developing brain. Thus, evaluating the fusion of multi-source 3D constructs is warranted for generating more anatomically and functionally representative human brain models. Another key limitation is that 3D constructs are cultured separately before fusion, resulting in assembloids that lack the native extracellular cues that guide neurodevelopment (Table 1). Future work may incorporate guided dynamic cocultures of 3D constructs in microfluidic platforms that separately mature and geometrically constrain these constructs before barrier removal for assembloid fusion (Cassel de Camps et al., 2024). Such systems systematically control geometry, cellular maturation, and natural cellular interactions, contributing to the realistic recapitulation of the human brain.

Taken together, these considerations underscore the need for continued ethical, design, developmental, and conceptual evaluation as assembloid platforms gain increasing biological complexity. As these systems approach higher-order integration, they not only expand experimental capability but also compel careful reflection on the boundaries between modeling neural function and approximating sentient biological states.

## References

[B1] Ahn Y. Chang H. Baek J. (2024). 3D scaffolds-specific cellular mechanoresponse as a pivotal regulating factor in tissue engineering. *JMST Adv.* 6 121–134. 10.1007/s42791-024-00076-y

[B2] Andersen J. Revah O. Miura Y. Thom N. Amin N. D. Kelley K. W. et al. (2020). Generation of functional human 3D cortico-motor assembloids. *Cell* 183 1913–1929.e26. 10.1016/j.cell.2020.11.017 33333020 PMC8711252

[B3] Bagley J. A. Reumann D. Bian S. Lévi-Strauss J. Knoblich J. A. (2017). Fused cerebral organoids model interactions between brain regions. *Nat. Methods* 14 743–751. 10.1038/nmeth.4304 28504681 PMC5540177

[B4] Birey F. Andersen J. Makinson C. D. Islam S. Wei W. Huber N. et al. (2017). Assembly of functionally integrated human forebrain spheroids. *Nature* 545 54–59. 10.1038/nature22330 28445465 PMC5805137

[B5] Birey F. Li M. Y. Gordon A. Thete M. V. Valencia A. M. Revah O. et al. (2022). Dissecting the molecular basis of human interneuron migration in forebrain assembloids from Timothy syndrome. *Cell Stem Cell* 29 248–264.e7. 10.1016/j.stem.2021.11.011 34990580 PMC13492730

[B6] Boroviak T. Stirparo G. G. Dietmann S. Hernando-Herraez I. Mohammed H. Reik W. et al. (2018). Single cell transcriptome analysis of human, marmoset and mouse embryos reveals common and divergent features of preimplantation development. *Development* 145:dev167833. 10.1242/dev.167833 30413530 PMC6240320

[B7] Cainelli E. Bisiacchi P. (2022). Neurodevelopmental disorders: Past, present, and future. *Children* 10:31. 10.3390/children10010031 36670582 PMC9856894

[B8] Cassel de Camps C. Rostami S. Xu V. Li C. Lépine P. Durcan T. M. et al. (2024). Microfabricated dynamic brain organoid cocultures to assess the effects of surface geometry on assembloid formation. *Biotechnol. J.* 19:e2400070. 10.1002/biot.202400070 39167558

[B9] Castagnola V. Deleye L. Podestà A. Jaho E. Loiacono F. Debellis D. et al. (2023). Interactions of graphene oxide and few-layer graphene with the blood-brain barrier. *Nano Lett.* 23 2981–2990. 10.1021/acs.nanolett.3c00377 36917703 PMC10103300

[B10] Chen A. Guo Z. Fang L. Bian S. (2020). Application of fused organoid models to study human brain development and neural disorders. *Front. Cell. Neurosci.* 14:133. 10.3389/fncel.2020.00133 32670022 PMC7326106

[B11] Chen H. Liang Y. Sun X. Xiong W. Yang T. Liang Y. et al. (2025). Generation of vascularized retinal organoids containing microglia based on a PDMS microwell platform. *Sci. Adv.* 11:eady6410. 10.1126/sciadv.ady6410 41071880 PMC12513421

[B12] Croxford J. Bayne T. (2024). The case against organoid consciousness. *Neuroethics* 17:13. 10.1007/s12152-024-09548-3

[B13] Damianidou E. Mouratidou L. Kyrousi C. (2022). Research models of neurodevelopmental disorders: The right model in the right place. *Front. Neurosci.* 16:1031075. 10.3389/fnins.2022.1031075 36340790 PMC9630472

[B14] Dao L. You Z. Lu L. Xu T. Sarkar A. K. Zhu H. et al. (2024). Modeling blood-brain barrier formation and cerebral cavernous malformations in human PSC-derived organoids. *Cell Stem Cell* 31 818–833.e11. 10.1016/j.stem.2024.04.019 38754427 PMC11162335

[B15] Dehorter N. Del Pino I. (2020). Shifting developmental trajectories during critical periods of brain formation. *Front. Cell. Neurosci.* 14:283. 10.3389/fncel.2020.00283 33132842 PMC7513795

[B16] Deng L. Dourado M. Reese R. M. Huang K. Shields S. D. Stark K. L. et al. (2023). Nav1.7 is essential for nociceptor action potentials in the mouse in a manner independent of endogenous opioids. *Neuron* 111 2642–2659.e13. 10.1016/j.neuron.2023.05.024 37352856

[B17] Dias D. Portugal C. C. Relvas J. Socodato R. (2025). From genetics to neuroinflammation: The impact of ApoE4 on microglial function in alzheimer’s disease. *Cells* 14:243. 10.3390/cells14040243 39996715 PMC11853365

[B18] Fligor C. M. Lavekar S. S. Harkin J. Shields P. K. VanderWall K. B. Huang K. C. et al. (2021). Extension of retinofugal projections in an assembled model of human pluripotent stem cell-derived organoids. *Stem Cell Rep.* 16 2228–2241. 10.1016/j.stemcr.2021.05.009 34115986 PMC8452489

[B19] Florio M. Huttner W. B. (2014). Neural progenitors, neurogenesis and the evolution of the neocortex. *Development* 141 2182–2194. 10.1242/dev.090571 24866113

[B20] Gentleman E. Dee K. C. Livesay G. A. Nauman E. A. (2007). Operating curves to characterize the contraction of fibroblast-seeded collagen gel/collagen fiber composite biomaterials: Effect of fiber mass. *Plast. Reconstr. Surg.* 119 508–516. 10.1097/01.prs.0000246316.87802.b4 17230083

[B21] Gentleman E. Nauman E. A. Dee K. C. Livesay G. A. (2004). Short collagen fibers provide control of contraction and permeability in fibroblast-seeded collagen gels. *Tissue Eng.* 10 421–427. 10.1089/107632704323061780 15165459

[B22] Hardy J. Selkoe D. J. (2002). The amyloid hypothesis of alzheimer’s disease: Progress and problems on the road to therapeutics. *Science* 297 353–356. 10.1126/science.1072994 12130773

[B23] Hayes M. H. Valdes Michel M. F. Kuehn M. H. Kardon R. H. Gramlich O. W. (2026). Generation of xenobiotic free retinofugal assembloids. *Front. Cell. Dev. Biol.* 13:1746709. 10.3389/fcell.2025.1746709 41613946 PMC12847054

[B24] Kanton S. Pasça S. P. (2022). Human assembloids. *Development* 149:dev.201120. 10.1242/dev.201120 36317797

[B25] Kataoka M. Niikawa T. Nagaishi N. Lee T. L. Erler A. Savulescu J. et al. (2025). Beyond consciousness: Ethical, legal, and social issues in human brain organoid research and application. *Eur. J. Cell Biol.* 104:151470. 10.1016/j.ejcb.2024.151470 39729735

[B26] Kim J. I. Imaizumi K. Jurjuţ O. Kelley K. W. Wang D. Thete M. V. (2025). Human assembloid model of the ascending neural sensory pathway. *Nature* 642 143–153. 10.1038/s41586-025-08808-3 40205039 PMC12137141

[B27] Kim J. I. Miura Y. Li M. Y. Revah O. Selvaraj S. Birey F. et al. (2024). Human assembloids reveal the consequences of CACNA1G gene variants in the thalamocortical pathway. *Neuron* 112 4048–4059.e7. 10.1016/j.neuron.2024.09.020 39419023

[B28] Kim J. Kim R. Lee W. Kim G. H. Jeon S. Lee Y. J. et al. (2025). Assembly of glioblastoma tumoroids and cerebral organoids: A 3D in vitro model for tumor cell invasion. *Mol. Oncol.* 19 698–715. 10.1002/1878-0261.13740 39473365 PMC11887666

[B29] Kyrousi C. O’Neill A. C. Brazovskaja A. He Z. Kielkowski P. Coquand L. et al. (2021). Extracellular LGALS3BP regulates neural progenitor position and relates to human cortical complexity. *Nat. Commun.* 12:6298. 10.1038/s41467-021-26447-w 34728600 PMC8564519

[B30] Lavazza A. (2020). Human cerebral organoids and consciousness: A double-edged sword. *Monash Bioeth. Rev.* 38 105–128. 10.1007/s40592-020-00116-y 32895775 PMC7723930

[B31] Lee J. Kim Y. Lee C. Jeon S. S. Seo H. Lee J. et al. (2025). Generation of prostate cancer assembloids modeling the patient-specific tumor microenvironment. *PLoS Genet.* 21:e1011652. 10.1371/journal.pgen.1011652 40163511 PMC12002641

[B32] Leung C. Jia Z. (2016). Mouse genetic models of human brain disorders. *Front Genet.* 7:40. 10.3389/fgene.2016.00040 27047540 PMC4803727

[B33] Lewus K. E. Nauman E. A. (2005). In vitro characterization of a bone marrow stem cell-seeded collagen gel composite for soft tissue grafts: Effects of fiber number and serum concentration. *Tissue Eng.* 11 1015–1022. 10.1089/ten.2005.11.1015 16144437

[B34] Li E. Benitez C. Boggess S. C. Koontz M. Rose I. V. L. Martinez D. et al. (2025). CRISPRi-based screens in iAssembloids to elucidate neuron-glia interactions. *Neuron* 113 701–718.e8. 10.1016/j.neuron.2024.12.016 39814010 PMC11886924

[B35] Li W. Li J. Y. (2024). Overlaps and divergences between tauopathies and synucleinopathies: A duet of neurodegeneration. *Transl. Neurodegener.* 13:16. 10.1186/s40035-024-00407-y 38528629 PMC10964635

[B36] Li W. Pozzo-Miller L. (2020). Dysfunction of the corticostriatal pathway in autism spectrum disorders. *J. Neurosci. Res.* 98 2130–2147. 10.1002/jnr.24560 31758607 PMC7242149

[B37] Liaudanskaya V. Fiore N. J. Zhang Y. Milton Y. Kelly M. F. Coe M. et al. (2023). Mitochondria dysregulation contributes to secondary neurodegeneration progression post-contusion injury in human 3D in vitro triculture brain tissue model. *Cell Death Dis.* 14:496. 10.1038/s41419-023-05980-0 37537168 PMC10400598

[B38] Liu Y. Zhou F. Ali H. Lathia J. D. Chen P. (2024). Immunotherapy for glioblastoma: Current state, challenges, and future perspectives. *Cell. Mol. Immunol.* 21 1354–1375. 10.1038/s41423-024-01226-x 39406966 PMC11607068

[B39] Maity S. Bhuyan T. Jewell C. Kawakita S. Sharma S. Nguyen H. T. et al. (2025). Recent developments in glioblastoma-on-a-chip for advanced drug screening applications. *Small* 21:e2405511. 10.1002/smll.202405511 39535474 PMC11719323

[B40] Makrygianni E. A. Chrousos G. P. (2021). From brain organoids to networking assembloids: Implications for neuroendocrinology and stress medicine. *Front. Physiol.* 12:621970. 10.3389/fphys.2021.621970 34177605 PMC8222922

[B41] McCrimmon C. M. Toker D. Pahos M. Cao Q. Lozano K. Lin J. J. et al. (2025). Cortical versus hippocampal network dysfunction in a human brain assembloid model of epilepsy and intellectual disability. *Cell Rep.* 44:116217. 10.1016/j.celrep.2025.116217 40925365 PMC12674601

[B42] Meng X. Yao D. Imaizumi K. Chen X. Kelley K. W. Reis N. et al. (2023). Assembloid CRISPR screens reveal impact of disease genes in human neurodevelopment. *Nature* 622 359–366. 10.1038/s41586-023-06564-w 37758944 PMC10567561

[B43] Meyer-Acosta K. K. Diaz-Guerra E. Varma P. Aruk A. Mirsadeghi S. Muniz-Perez A. et al. (2025). APOE4 impacts cortical neurodevelopment and alters network formation in human brain organoids. *Stem Cell Rep.* 20:102537. 10.1016/j.stemcr.2025.102537 40541173 PMC12277819

[B44] Miura Y. Li M. Y. Birey F. Ikeda K. Revah O. Thete M. V. et al. (2020). Generation of human striatal organoids and cortico-striatal assembloids from human pluripotent stem cells. *Nat. Biotechnol.* 38 1421–1430. 10.1038/s41587-020-00763-w 33273741 PMC9042317

[B45] Nakajima M. Schmitt L. I. (2020). Understanding the circuit basis of cognitive functions using mouse models. *Neurosci. Res.* 152 44–58. 10.1016/j.neures.2019.12.009 31857115

[B46] Onesto M. M. Amin N. D. Pan C. Chen X. Kim J. I. Reis N. (2025). Midline assembloids reveal regulators of human axon guidance. *Science* 389 282–289. 10.1126/science.adq7934 40674484 PMC12788182

[B47] Onesto M. M. Kim J. I. Pasca S. P. (2024). Assembloid models of cell-cell interaction to study tissue and disease biology. *Cell Stem Cell* 31 1563–1573. 10.1016/j.stem.2024.09.017 39454582 PMC12143640

[B48] Ouaidat S. Bellapianta A. Ammer-Pickhardt F. Taghipour T. Bolz M. Salti A. (2025). Exploring organoid and assembloid technologies: A focus on retina and brain. *Expert Rev. Mol. Med.* 27:e14. 10.1017/erm.2025.9 40145178 PMC12011387

[B49] Paulson H. L. Shakkottai V. G. Clark H. B. Orr H. T. (2017). Polyglutamine spinocerebellar ataxias — from genes to potential treatments. *Nat. Rev. Neurosci.* 18 613–626. 10.1038/nrn.2017.92 28855740 PMC6420820

[B50] Pipicelli F. Baumann N. Di Giaimo R. Forero-Echeverry A. Kyrousi C. Bonrath R. et al. (2023). Non–cell-autonomous regulation of interneuron specification mediated by extracellular vesicles. *Sci. Adv.* 9:eadd8164. 10.1126/sciadv.add8164 37205765 PMC10198641

[B51] Poblano J. Castillo-Tobías I. Berlanga L. Tamayo-Ordoñez M. C. del Carmen Rodríguez-Salazar M. Silva-Belmares S. Y. (2024). Drugs targeting APOE4 that regulate beta-amyloid aggregation in the brain: Therapeutic potential for Alzheimer’s disease. *Basic Clin. Pharmacol. Toxicol.* 135 237–249. 10.1111/bcpt.14055 39020526

[B52] Roy D. S. Zhang Y. Aida T. Choi S. Chen Q. Hou Y. et al. (2021). Anterior thalamic dysfunction underlies cognitive deficits in a subset of neuropsychiatric disease models. *Neuron* 109 2590–2603.e13. 10.1016/j.neuron.2021.06.005 34197733 PMC8376805

[B53] Saili K. S. Zurlinden T. J. Schwab A. J. Silvin A. Baker N. C. Hunter I. I. I. E. S. et al. (2017). Blood-brain barrier development: Systems modeling and predictive toxicology. *Birth Defects Res.* 109 1680–1710. 10.1002/bdr2.1180 29251840 PMC6476421

[B54] Samarasinghe R. A. Miranda O. A. Buth J. E. Mitchell S. Ferando I. Watanabe M. et al. (2021). Identification of neural oscillations and epileptiform changes in human brain organoids. *Nat. Neurosci.* 24 1488–1500. 10.1038/s41593-021-00906-5 34426698 PMC9070733

[B55] Shepherd G. M. G. (2013). Corticostriatal connectivity and its role in disease. *Nat. Rev. Neurosci.* 14 278–291. 10.1038/nrn3469 23511908 PMC4096337

[B56] Shin D. Kim C. N. Ross J. Hennick K. M. Wu S. R. Paranjape N. et al. (2024). Thalamocortical organoids enable *in vitro* modeling of 22q11.2 microdeletion associated with neuropsychiatric disorders. *Cell Stem Cell* 31 421–432.e8. 10.1016/j.stem.2024.01.010 38382530 PMC10939828

[B57] Sinha S. Huang M. S. Mikos G. Bedi Y. Soto L. Lensch S. et al. (2024). Laminin-associated integrins mediate Diffuse Intrinsic Pontine Glioma infiltration and therapy response within a neural assembloid model. *Acta Neuropathol. Commun.* 12:71. 10.1186/s40478-024-01765-4 38706008 PMC11070088

[B58] Son J. Park S. J. Ha T. Lee S. N. Cho H. Y. Choi J. W. (2022). Electrophysiological monitoring of neurochemical-based neural signal transmission in a human brain-spinal cord assembloid. *ACS Sens.* 7 409–414. 10.1021/acssensors.1c02279 35044765

[B59] Sydnor V. J. Bagautdinova J. Larsen B. Arcaro M. J. Barch D. M. Bassett D. S. et al. (2025). Human thalamocortical structural connectivity develops in line with a hierarchical axis of cortical plasticity. *Nat. Neurosci.* 28 1772–1786. 10.1038/s41593-025-01991-6 40615590 PMC12321582

[B60] Tran H. D. Shin M. K. Yeo X. Y. Jung S. Junaid M. Lim S. B. et al. (2025). A human striatal-midbrain assembloid model of alpha-synuclein propagation. *Brain* 149 867–883. 10.1093/brain/awaf326 40919647

[B61] Walsh R. M. Crabtree G. W. Kalpana K. Jubierre L. Koo S. Y. Ciceri G. et al. (2025). Forebrain assembloids support the development of fast-spiking human PVALB+ cortical interneurons and uncover schizophrenia-associated defects. *Neuron* 113 3185–3203.e7. 10.1016/j.neuron.2025.06.017 40695284 PMC12447774

[B62] Walsh R. M. Luongo R. Giacomelli E. Ciceri G. Rittenhouse C. Verrillo A. et al. (2024). Generation of human cerebral organoids with a structured outer subventricular zone. *Cell Rep.* 43:114031. 10.1016/j.celrep.2024.114031 38583153 PMC11322983

[B63] Wu S. R. Nowakowski T. J. (2025). Exploring human brain development and disease using assembloids. *Neuron* 113 1133–1150. 10.1016/j.neuron.2025.02.010 40107269 PMC12022838

[B64] Wu S. Hong Y. Chu C. Gan Y. Li X. Tao M. et al. (2024). Construction of human 3D striato-nigral assembloids to recapitulate medium spiny neuronal projection defects in Huntington’s disease. *Proc. Natl. Acad. Sci. U.S.A.* 121:e2316176121. 10.1073/pnas.2316176121 38771878 PMC11145230

[B65] Xiang Y. Tanaka Y. Patterson B. Kang Y. J. Govindaiah G. Roselaar N. et al. (2017). Fusion of regionally specified hPSC-derived organoids models human brain development and interneuron migration. *Cell Stem Cell* 21 383–398.e7. 10.1016/j.stem.2017.07.007 28757360 PMC5720381

[B66] Xu B. Lei X. Yang Y. Yu J. Chen J. Xu Z. et al. (2025). Peripheral proteinopathy in neurodegenerative diseases. *Transl. Neurodegener.* 14:2. 10.1186/s40035-024-00461-6 39819742 PMC11737199

[B67] Zhang X. Zhao Y. Mao L. Wang Q. Huang P. (2026). Mechanochemical regulation of organoid morphogenesis: Integrated signaling circuits in engineered microenvironments. *Materials Today Bio* 37:102782. 10.1016/j.mtbio.2026.102782 41585444 PMC12828605

[B68] Zhang Y. Savvidou M. Liaudanskaya V. Ramanathan V. Bui T. Lindley M. et al. (2025). Multi-modal label-free imaging of cellular metabolism and oxidative stress in 3D brain tissue models. *Commun. Biol.* 8:1737. 10.1038/s42003-025-09122-4 41331326 PMC12673076

